# Significance of Cardiac Troponin in Cancer Patients

**DOI:** 10.3390/cells15161435

**Published:** 2026-08-10

**Authors:** Kristóf Birgés, Béla Merkely, Andrea Ágnes Molnár

**Affiliations:** Heart and Vascular Center, Semmelweis University, 1122 Budapest, Hungary; birges.kristof@stud.semmelweis.hu (K.B.); merkely.bela@gmail.com (B.M.)

**Keywords:** troponin, cardio-oncology, cancer

## Abstract

Recent advances in oncological therapies have significantly improved cancer survival rates. However, these therapies have also led to an increased risk of cardiovascular alterations. Cardiac troponin, which is well-known for its role in diagnosing myocardial damage, is gaining recognition as a biomarker in the field of cardio-oncology. Despite its clinical value, differential diagnosis remains challenging, as troponin levels can increase in numerous scenarios, particularly in cardio-oncology. While the most recognized cause is the cardiotoxicity associated with cancer therapy, malignant tumors may also directly impact the myocardium. Furthermore, different cardiac troponins, such as troponins T and I, may have distinct biomarker roles, adding to diagnostic complexity. This review aims to summarize the pathological conditions that cause elevated serum cardiac troponin levels in patients with malignancies. We explore the underlying mechanisms at the molecular and cellular levels and discuss potential clinical applications. We also highlight existing gaps in the current evidence and outline future research directions in this rapidly evolving field.

## 1. Introduction

Cancer remains one of the leading causes of morbidity and mortality worldwide [1]. While advances in oncological therapy now offer long-term survival to an increasing number of patients, both the disease itself and anti-cancer treatments can adversely affect the cardiovascular system through multiple pathways. These include therapy-induced myocardial damage, the direct or indirect effects of the tumor, and shared cardiovascular risk factors or molecular mechanisms [2,3,4]. Notably, cardiovascular disease is the second leading cause of morbidity and mortality in cancer survivors, surpassed only by recurrent malignancy [5]. Because cardiotoxicity significantly impairs overall prognosis, specialized cardio-oncology care plays a pivotal role in managing this patient population [3].

Cardiac biomarkers, such as troponins, are established indicators of myocardial damage. They are structural components of the troponin–tropomyosin complex within cardiomyocytes and are released into the circulation upon cardiac injury. However, their limited etiological specificity poses significant differential diagnostic challenges, particularly within the oncological population [6,7]. In cancer patients, the role of troponin extends far beyond the diagnosis of acute myocardial infarction. It serves as a dynamic tool for early detection, risk stratification, monitoring treatment complications, and prognostic assessment in cancer patients according to the 2022 European Society of Cardiology (ESC) Guidelines on Cardio-Oncology [8]. They recommend routine monitoring of cardiac troponin levels in patients undergoing cardiotoxic therapies, such as anthracyclines, human epidermal growth factor receptor (HER) 2 inhibitors, and immune checkpoint inhibitors [8]. Furthermore, cardiological surveillance for patients outside high-risk categories remains less defined in current guidelines and standard cardio-oncological care [9]. Recent research suggests that circulating troponin levels may possess prognostic value across a broader spectrum of cancer patients [7,9,10,11]. Given its widespread availability, troponin could serve as a first-line marker for screening and risk stratification regarding treatment-induced cardiotoxicity [12]. Ultimately, an integrated approach combining biomarkers, clinical parameters, and imaging modalities represents the most promising strategy for enhancing diagnostic accuracy and patient management [13]. Nevertheless, there is a lack of consensus regarding optimal cut-off values, specificity, and sensitivity of troponin in cancer patients [14].

Despite 30 years of diagnostic use, troponin’s role in oncology is not yet fully established. Given the rising number of cancer survivors, optimizing cardio-oncological care using precise troponin assessment is essential. Currently, its clinical application—specifically regarding timing, cut-off values, and its role as an early warning signal, safety monitor, or prognosticator—is limited to only a few oncological agents. This review explores the cellular, molecular, and pathophysiological mechanisms of troponin release, as well as its prognostic value for cardiotoxicity and survival in cancer patients.

## 2. Cardiac Troponin Physiology and Diagnostic Use

**Figure 1:**
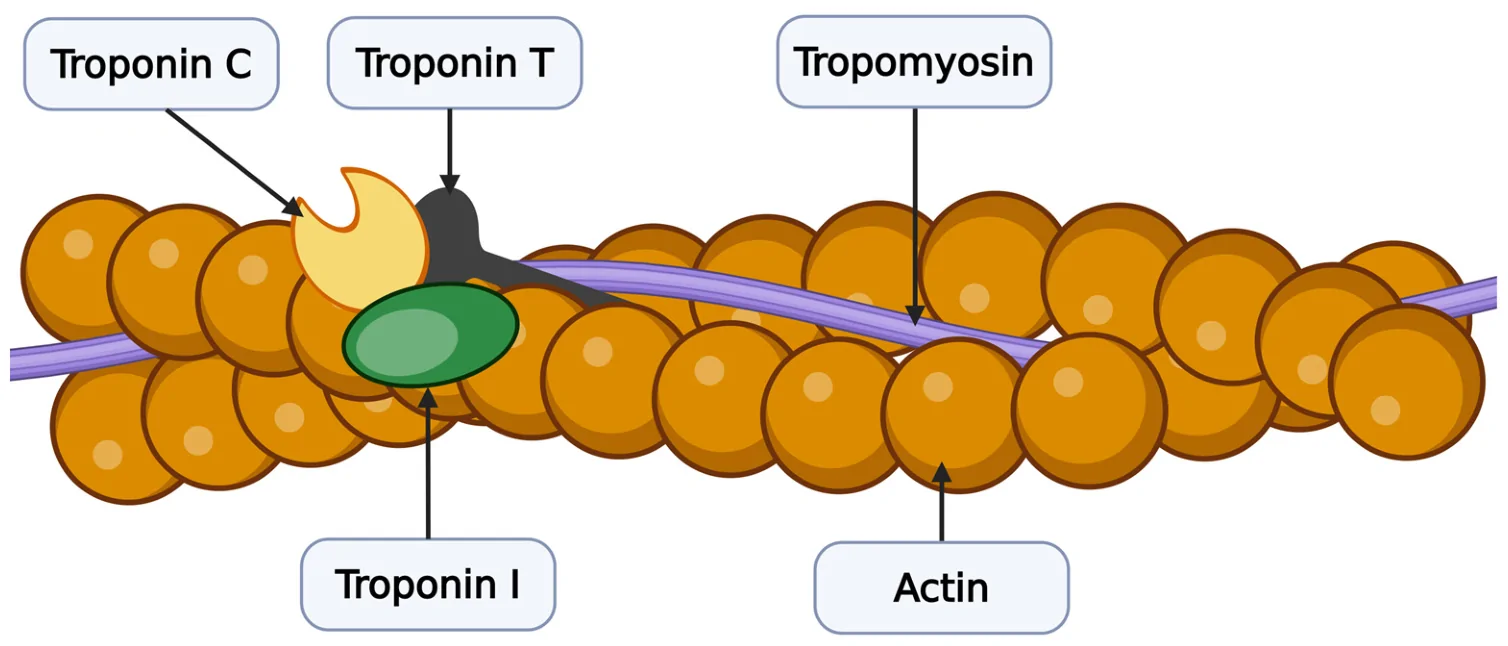
Schematic illustration of the thin filament: the troponin complex, tropomyosin, and actin. The different shapes of troponin T, I, and C are intended to illustrate that they are distinct molecules with different functions and do not represent their accurate three-dimensional structures. (Created in BioRender. Birgés, K. (2026) https://BioRender.com/stc0ipz).

Cardiac muscle is composed of cardiomyocytes, which contain contractile units called sarcomeres. While sarcomeres in cardiac tissue share similarities with those in skeletal muscle, they also exhibit certain differences. Central to their function is the troponin complex, a regulatory unit consisting of three subunits: cardiac troponin C (cTnC), cardiac troponin I (cTnI), and cardiac troponin T (cTnT), which, together with tropomyosin and actin, forms the thin filament of the sarcomere (Figure 1). cTnT, encoded by the TNNT2 gene, is responsible for binding tropomyosin, while cTnI, encoded by the TNNI3 gene, functions as the inhibitory subunit in cardiomyocytes. During intrauterine development, cardiomyocytes express a non-cardiac isoform of troponin I, whereas fetal skeletal muscles transiently express the cardiac isoform of troponin T. In adulthood, however, cardiomyocytes and skeletal muscle cells strictly express their respective tissue-specific isoforms under physiological conditions. Both cTnT and cTnI possess distinct amino acid sequences absent in the skeletal isoforms of troponin T and I, rendering them highly specific targets for the diagnosis of myocardial injury. Conversely, troponin C, encoded by the TNNC1 gene, serves as the calcium-binding component of the complex but is expressed in both slow-twitch skeletal and cardiac muscle, whereas a distinct isoform is expressed in fast-twitch skeletal muscle. Since it lacks cardiac specificity, troponin C is not utilized in the diagnosis of myocardial injury [15,16,17].

Following myocardial injury, both cTnT and cTnI can be detected in blood; however, their kinetics differ. cTnI generally demonstrates a greater early rise following acute myocardial injury, whereas cTnT exhibits a more prolonged release pattern and persists elevated for a longer period after injury. Moreover, cTnT levels are more strongly influenced by comorbidities, such as diabetes mellitus and renal function, with reduced renal function resulting in slower cTnT clearance [18,19,20]. While cTnT and cTnI show different predictive values for cardiovascular outcomes [18], limited evidence is available comparing their performance in cardiotoxicity [21]. A significant differential diagnostic challenge arises from cTnT positivity in the setting of skeletal muscle diseases. This phenomenon is driven either by the re-expression of cTnT isoforms within diseased skeletal muscle tissue or by analytical cross-reactivity between skeletal muscle troponin T and cTnT assays [22]. As a result, this can lead to diagnostic difficulties in cancer patients with immune checkpoint inhibitor (ICI)-induced myositis who lack myocardial involvement. In these scenarios, cTnT levels can be markedly elevated while cTnI remains within normal limits; consequently, relying solely on cTnT testing may cause a misdiagnosis of concomitant myocarditis [23,24,25].

The majority of cardiac troponin I and T is structurally integrated into the troponin–tropomyosin complex of the thin filaments, while a smaller, unbound fraction resides within the cytoplasm, representing approximately 5% of cTnI and 8% of cTnT. This cytosolic reserve is clinically significant because it underlies the rapid, early release of biomarkers following cardiomyocyte membrane injury [16]. Furthermore, recent evidence indicates that troponin also localizes within the nucleus, where it may function as a regulatory protein [26].

**Figure 2:**
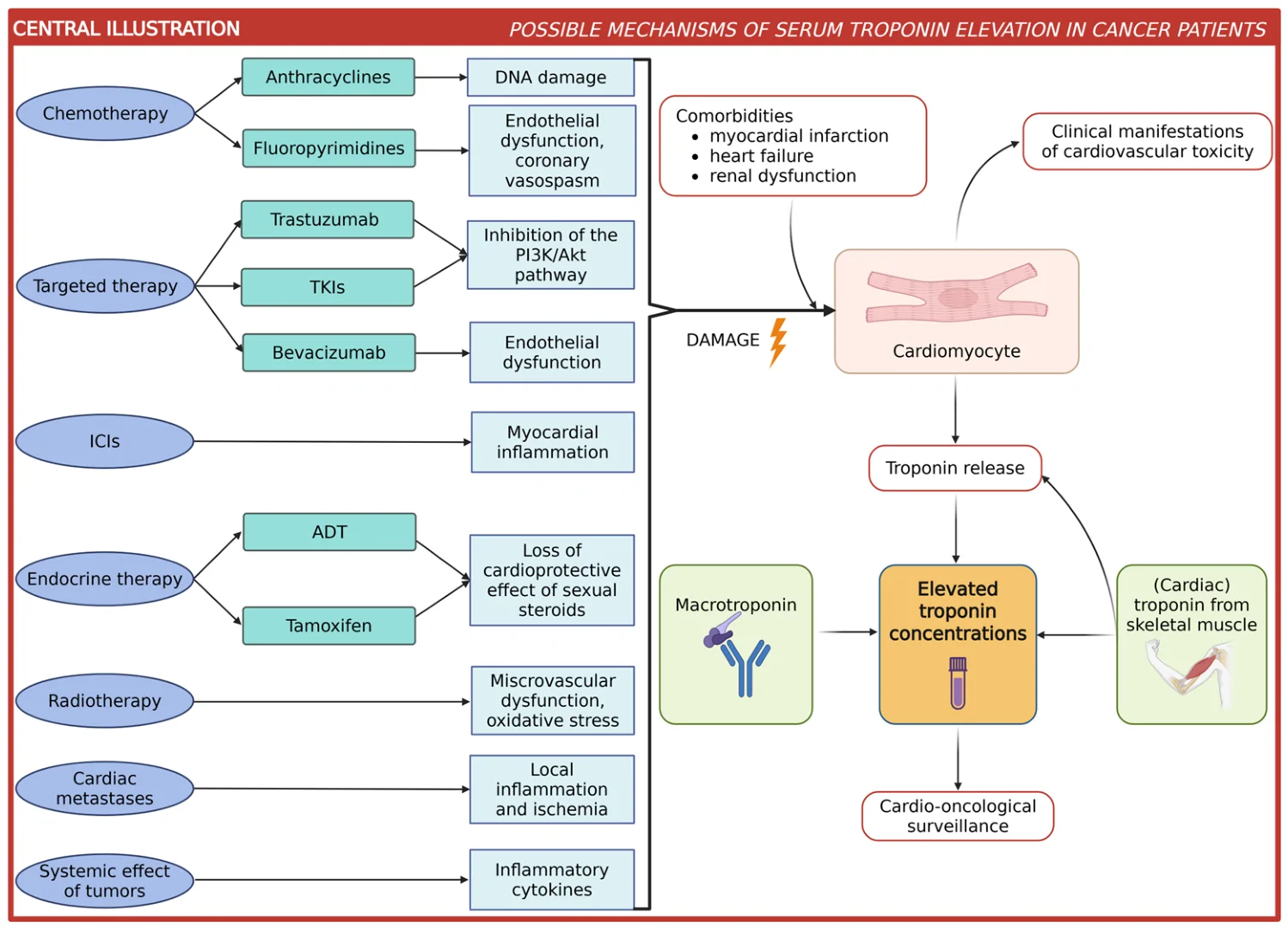
Central illustration. Possible mechanisms of serum troponin elevation in cancer patients. Antineoplastic agents induce myocardial injury through distinct mechanisms. Chemotherapeutic agents (e.g., anthracyclines and fluoropyrimidines) cause direct DNA damage or ischemia through vascular toxicity. Targeted therapies, including trastuzumab and tyrosine kinase inhibitors, inhibit the phosphatidylinositol 3-kinase/Akt pathway, whereas bevacizumab induces endothelial dysfunction. Immune checkpoint inhibitors promote myocardial inflammation, while endocrine therapies (androgen deprivation therapy and tamoxifen) exert cardiotoxic effects through sex steroid depletion or antagonism. Chest irradiation causes myocardial microvascular dysfunction and oxidative stress. Cancer itself may also affect the heart through local inflammation, ischemia secondary to cardiac metastases, or tumor-derived inflammatory cytokines. Together with comorbidities, these mechanisms contribute to cardiomyocyte injury and subsequent cardiac troponin release. Troponin may also originate from diseased skeletal muscle, whereas assay interference by macrotroponin or excess skeletal muscle troponin can result in false-positive elevations. Clinically, cardiac troponin measurement is an integral component of cardio-oncology surveillance. Importantly, the mechanisms described not only lead to troponin elevation, but also contribute to the development of clinical symptoms of cardiotoxicity. Abbreviations: TKIs: tyrosine kinase inhibitors; ICIs: immune checkpoint inhibitors; ADT: androgen deprivation therapy; PI3K: phosphatidylinositol 3-kinase. (Created in BioRender. Birgés, K. (2026) https://BioRender.com/mw6std2).

Measurement of cardiac troponins in peripheral blood is a cornerstone in the diagnosis of acute myocardial infarction. The widespread implementation of high-sensitivity (hs) cardiac troponin assays has enabled the detection of trace concentrations of circulating cardiac troponin isoforms; however, this enhanced analytical sensitivity may compromise clinical specificity. Troponin release is no longer observed exclusively during massive cardiomyocyte necrosis—which produces marked elevations in serum levels—but also during minor myocardial injury, resulting in subtle troponin elevations that are now quantifiable via high-sensitivity assays [16,27]. These high-sensitivity immunoassays capture troponin molecules using specific antibodies to generate a quantifiable signal [28]. cTnT testing relies predominantly on a single globally standardized assay, whereas multiple assay systems are employed for cTnI measurement [29]. These assays differ significantly in their analytical sensitivity—with several cTnI platforms capable of detecting lower concentrations than the cTnT assay—as well as in their epitope specificity and susceptibility to analytical interference [19]. Consequently, serial monitoring of troponin levels in cardio-oncology patients must be performed consistently using the same assay platform to ensure clinical comparability [28,30]. Another clinically relevant aspect of troponin interpretation during cardiotoxicity monitoring is its elimination half-life, which has been reported to be approximately 134 min for cTnT and 240 min for cTnI in a recent study [31]. Aberrant results, including false positives and, less frequently, false negatives, can arise from cross-reacting molecules or analytical interferences. These include heterophilic antibodies, fibrin clots, skeletal-muscle-derived TnT, and macrotroponin [19,28]. Macrotroponin is a circulating immune complex consisting of one or more troponin molecules bound to troponin-specific autoantibodies. Due to its reduced clearance—and therefore prolonged half-life—compared to free troponin, macrotroponin can cause persistently elevated troponin levels in the absence of acute myocardial injury. Although the presence of macrotroponin in oncological patients has been documented, the literature on its significance remains scarce, and further studies are warranted to determine the extent to which macrotroponin confounds differential diagnosis in this cohort [28,32,33] (Figure 2).

## 3. Molecular and Cellular Mechanisms of Troponin Release from Cardiomyocytes

*Necrosis* due to myocardial infarction is the traditionally well-described cause for troponin release; however, other etiologies also exist, such as direct physical trauma or toxins. Occlusion of a coronary artery results in myocardial hypoxia, leading to the production of reactive oxygen species (ROS), activation of certain proteases, such as Ca^2+^-dependent calpain and cathepsins, and cellular swelling. Eventually, cardiomyocyte cell membrane rupture occurs, releasing free cytoplasmic cTnT and cTnI into the circulation, followed by troponins from the thin filament after proteolytic degradation [15,16]. Apart from necrosis, cell death can occur in regulated pathways, such as apoptosis, necroptosis, pyroptosis and ferroptosis.

*Apoptosis* may significantly contribute to the release of cardiac troponins into the systemic circulation due to short-term ischemia, pathological increases in myocardial wall stretch, and chronic neurohormonal stimulation via β-adrenergic receptors. This pathway is characterized by an upregulation of caspase activity (for instance, caspase-8 and caspase-3), proteases that catalyze the degradation of chromosomal DNA and cellular proteins, including troponins. Troponin subunits are not immediately released into the interstitial space; instead, they remain sequestered within apoptotic bodies. These membrane-bound vesicles are typically destined for phagocytosis and elimination by macrophages. However, it has been proposed that failure of vesicle closure or disruption of apoptotic bodies can permit the release of troponin fragments into the interstitial space and ultimately into the systemic circulation [15,16,34,35].

*Necroptosis* is involved in doxorubicin-induced cardiotoxicity. In this process, immune ligands including tumor necrosis factor (TNF) and Fas trigger activation of receptor-interacting protein kinases 1/3, leading to downstream activation of mixed-lineage kinase domain-like protein 1, which forms multimers and binds to the cell membrane. This creates pores that eventually lead to cell lysis. It is hypothesized that troponin is released during this lysis, contributing to elevated circulating troponin levels [36,37].

*Pyroptosis* is associated with cardiotoxicity induced by anthracyclines, trastuzumab, and ICIs [38,39,40]. Initially, ROS in cardiomyocytes trigger the formation of a protein complex called the nucleotide-binding oligomerization domain-like receptor family pyrin domain containing 3 inflammasome. This complex activates caspase-1, which promotes the maturation and release of interleukin (IL)-1β and IL-18. Gasdermin D is also activated and forms pores in the cell membrane, allowing leakage of cytoplasmic soluble proteins, including pro-inflammatory cytokines, into the extracellular space [38,41,42]. Hempel et al. demonstrated that there is no size limit for the intracellular soluble proteins released during pyroptosis, suggesting that troponin could also be released from cardiomyocytes via this mechanism [41]. Eventually, the cell membrane ruptures, releasing the remaining intracellular contents into the extracellular environment [42].

*Ferroptosis* is an iron-dependent form of regulated cell death. During this process, iron ions accumulate—often due to excessive autophagy—leading to lipid peroxidation [43]. Eventually, the cell membrane ruptures [44], allowing troponin release. Ferroptosis has been linked to ischemic myocardial injury and various anti-tumor agents, such as anthracyclines, etoposide, paclitaxel, and trastuzumab [45,46,47]. Experimental data indicate that anthracyclines reduce Beclin 1 expression and increase Hmox1 levels, both of which are pro-ferroptotic changes [43,46].

Specific inhibitors of regulated cell death pathways, administered simultaneously with anticancer agents, have reduced myocardial injury and measurably lowered released troponin levels in experimental studies [37,38,43].

## 4. Troponin Release and Cardiotoxic Oncological Therapy

### 4.1. Chemotherapy

Conventional chemotherapy remains a cornerstone of oncological treatment. However, it is well established that numerous cytotoxic agents exert clinically significant cardiotoxic effects—as indicated by elevated troponin levels—which may necessitate dose reduction and significantly compromise long-term outcomes [48].

*Anthracyclines.* Anthracycline-induced cardiotoxicity, typically dose-dependent, represents one of the most extensively studied models of chemotherapy-related cardiac injury and troponin release [49]. The underlying mechanisms are multifactorial. A central pathway involves interaction with topoisomerase II β, leading to DNA damage and subsequent cell death, as well as ROS production resulting from anthracycline metabolism [49,50]. Other mechanisms of cardiotoxicity include mitochondrial dysfunction caused by impaired iron regulation—resulting in further ROS generation—as well as interference with the respiratory chain and phosphate metabolism. These latter processes may eventually contribute to delayed cardiotoxicity. Furthermore, endothelial injury, disrupted vascular signaling, and impaired calcium-dependent cellular functions exacerbate the damage [49]. Collectively, these mechanisms result in cardiomyocyte injury and various forms of cell death—including apoptosis, pyroptosis, ferroptosis, and necroptosis—leading to troponin release [49,51]. Manifestations of anthracycline-induced cardiotoxicity include dose-dependent left ventricular dysfunction—typically presenting as a reduction in ejection fraction—as well as cardiomyopathy, heart failure, and arrhythmias [52]. Clinically, anthracycline exposure is characterized by dynamic elevations in cardiac troponin levels that often increase progressively over treatment cycles. Crucially, early elevations in hs-cardiac troponins have been shown to predict subsequent myocardial injury, highlighting their utility as sensitive biomarkers for subclinical cardiotoxicity [12,53,54]. Consequently, cardiac troponins have become central to the detection and monitoring of anthracycline-related injury [8].

*Fluoropyrimidines*, namely 5-fluorouracil and its oral prodrug capecitabine, are well-established cardiotoxic agents. While the precise mechanisms underlying their cardiotoxicity and associated troponin elevation remain to be fully elucidated, several hypotheses have been proposed based on current evidence. These involve direct injury to cardiomyocytes, mitochondrial impairment, vascular mechanisms, such as endothelial dysfunction and coronary vasospasm, and even disturbances in erythrocyte and iron homeostasis [55]. The diagnostic utility of troponin in fluoropyrimidine-induced cardiotoxicity remains controversial due to an inconsistent correlation between clinical symptoms and biomarker elevation. Zalaquett et al. investigated 137 patients, all of them with fluoropyrimidine-associated new-onset angina, and reported elevated troponin levels in 41.6% of cases [56]. Dyhl-Polk et al. studied 108 patients treated with 5-fluorouracil and observed acute coronary syndrome or new-onset arrhythmia in 7.4% of patients, while detectable troponin levels were present in 11.8%. However, no significant changes in troponin concentrations were observed during therapy compared with baseline values [57]. Similarly, Moghaddam et al. evaluated 100 cardiovascular disease-free patients receiving 5-fluorouracil and reported cardiotoxic events in 8% and detectable troponin levels in 15% of patients, including some without clinical symptoms. Nevertheless, they found no significant difference in the proportion of patients with detectable troponin levels before and after treatment initiation [58]. Another study also reported troponin elevation in a subset of patients without clinical signs or symptoms of cardiotoxicity; however, interpretation of these findings may be limited by the concomitant use of other potentially cardiotoxic therapies [58,59]. Collectively, these data underscore the need for larger, high-powered studies to establish more reliable diagnostic markers.

Other specific chemotherapy drugs, such as *alkylating agents* (e.g., *cyclophosphamide*, ifosfamide), *platinum-based drugs* (e.g., *cisplatin*), and *taxanes* (e.g., *paclitaxel*), are well-recognized cardiotoxic culprits [7,52]. Experimental studies in mice and cellular models have demonstrated that myocardial injury and subsequent troponin release are primarily driven by oxidative stress characterized by excessive ROS production, depletion of antioxidant defenses (such as glutathione, superoxide dismutase, catalase, glutathione peroxidase), and lipid peroxidation, ultimately leading to membrane disruption and troponin leakage. Oxidative stress activates inflammatory and apoptotic signaling pathways that amplify cardiac damage [47,60,61,62,63,64,65,66,67,68]. Additionally, in some cardiotoxicity models, ferroptosis and necrosis have also been observed as potential mechanisms of cell death that contribute to troponin release [47,64,69]. While troponins play a substantial role in diagnosing certain conditions induced by these agents, such as myocardial infarction [8], research is lacking regarding their role in predicting potential myocardial damage and survival.

### 4.2. Monoclonal Antibodies

*Anti-HER2 therapy.* Monoclonal antibodies targeting the HER2 receptor tyrosine kinase, including trastuzumab and pertuzumab, are widely used to treat breast and gastric cancers [70]. However, trastuzumab-associated cardiotoxicity remains a significant concern, affecting 12–17% of treated patients [71,72]. Although traditionally considered dose-independent and clinically reversible [12], recent evidence suggests that myocardial injury may be only partially reversible due to cardiomyocyte loss and ultrastructural damage [70,73,74]. HER2 is also expressed in cardiomyocytes, where it supports resistance to oxidative stress and cell survival through signaling pathways such as phosphatidylinositol 3-kinase (PI3K)/Akt, mitogen activated protein kinase, Janus kinase/signal transducer and activator of transcription (STAT) 3, Src/focal adhesion kinase, and endothelial nitric oxide synthase (eNOS). Inhibition of these pathways disrupts antioxidant defenses, promotes oxidative and nitrative stress, impairs mitochondrial function and calcium handling, and ultimately triggers cardiomyocyte death [12,70,74,75,76]. In addition to apoptosis, other forms of cell death, such as pyroptosis and ferroptosis, have also been described in experimental models and may contribute to myocardial troponin release [40].

Several studies have examined cardiac troponins as biomarkers of trastuzumab-related cardiotoxicity. In 78 breast cancer patients treated with anthracycline–cyclophosphamide followed by trastuzumab–taxane therapy, cTnI levels increased progressively during treatment, and concentrations measured after four cycles of trastuzumab–taxane predicted subsequent cardiotoxicity, with a proposed cut-off of 84 ng/L. However, the relative contribution of trastuzumab versus prior anthracycline exposure remains uncertain [77]. Similarly, Xia et al. found in 600 trastuzumab-treated breast cancer patients that cTnI elevation above the upper limit of normal independently predicted cardiovascular toxicity [78]. Cardinale et al. reported comparable findings in 251 patients, demonstrating that cTnI positivity was a strong independent predictor of cardiotoxicity. Importantly, all patients who developed cardiac dysfunction without troponin elevation experienced complete recovery, whereas recovery occurred in only 35% of those with elevated troponin levels. Based on this observation, the authors proposed that troponin measurements may predict the reversibility of trastuzumab-induced cardiotoxicity [79]. To date, however, this hypothesis has not been specifically investigated in subsequent studies.

*Anti-VEGF-A therapy.* Bevacizumab is a monoclonal antibody that targets vascular endothelial growth factor A (VEGF-A) and is widely used to treat breast, colorectal, renal, and other malignancies. By inhibiting tumor angiogenesis, bevacizumab suppresses tumor growth and metastatic spread. However, VEGF-A inhibition also reduces eNOS activity and increases endothelin production, promoting systemic and pulmonary hypertension [49,80,81]. Cardiovascular toxicity occurs in up to one-quarter of treated patients [80]. The mechanisms underlying bevacizumab-induced cardiotoxicity are multifactorial and include hypertension, endothelial injury, mitochondrial impairment, oxidative stress, endoplasmic reticulum stress, and inhibition of the extracellular signal-regulated kinase pathway [81]. Experimental studies suggest that bevacizumab has the potential to induce troponin release. Bordun et al. observed elevated serum cTnT levels in bevacizumab-treated mice, preceded by increased blood pressure. Troponin release was accompanied by loss of cellular integrity, myofibrillar disorganization, oxidative stress, inflammation, and enhanced apoptotic cell death, supporting a mechanistic link between bevacizumab-induced myocardial injury and circulating cardiac troponin elevation in this animal model [80]. Further insight into the underlying molecular mechanisms was provided by Chen et al., who demonstrated that bevacizumab induced apoptosis and cTnT release in HL-1 cardiomyocytes. The authors identified interferon regulatory factor-1, together with its downstream mediators VEGF-A and 14-3-3γ, as potential regulators of this injury pathway [81].

### 4.3. Tyrosine Kinase Inhibitors

Tyrosine kinase inhibitors (TKIs) are small-molecule agents that block tyrosine kinase activity, thereby inhibiting downstream oncogenic signaling pathways. Despite their clinical efficacy, TKIs are associated with significant cardiovascular toxicity [2,49,82]. A common mechanism of cardiotoxicity implicated across several TKIs, such as sunitinib, imatinib, and trametinib, is inhibition of PI3K signaling, leading to aberrant intracellular Ca^2+^ accumulation, increased ROS production, and impaired cardiomyocyte contractility [49,83].

Experimental and clinical studies provide evidence that TKI-induced myocardial injury may be accompanied by troponin release. Using an AC16 cardiomyocyte model, Gao et al. demonstrated that sunitinib, imatinib, and trametinib induced significant elevations in cTnI at their respective half-maximal inhibitory concentrations [83]. Similarly, Bouitbir et al. reported increased plasma cTnI levels and enhanced cardiomyocyte apoptosis in sunitinib-treated mice, while Alhoshani et al. observed significantly elevated serum troponin concentrations in rats exposed to gefitinib [82,84]. Clinical observations support these findings; in a cohort of 68 patients receiving sunitinib, Chu et al. detected asymptomatic cTnI elevations in 12 patients, although it remains unclear whether prior anthracycline exposure contributed to this finding [85].

Beyond inducing troponin release, TKIs may affect the cardiac contractile apparatus. Experimental studies have shown that sorafenib, sunitinib, and trametinib suppress TNNT2 expression, potentially contributing to impaired myocardial contractility [86]. Likewise, Ji et al. reported dysregulated expression of TNNT2, TNNC1, and TNNI3 in vandetanib-treated mice. In human induced pluripotent stem cell-derived cardiomyocytes, vandetanib disrupted the structural network of cardiac troponin T, cardiac troponin I, and α-actinin. These alterations suggest that TKI-induced cardiotoxicity involves perturbation of the cardiac contractile machinery [87].

### 4.4. Immune Checkpoint Inhibitors

The introduction of immunotherapy has revolutionized the treatment of numerous malignancies, markedly improving patient survival over the past decade. However, these therapeutic advances are accompanied by immune-related adverse events, including ICI-associated myocarditis [88]. Immune-mediated mechanisms are central to the pathogenesis of ICI cardiotoxicity. By blocking the inhibitory immune checkpoints cytotoxic T-lymphocyte-associated protein 4, programmed cell death protein (PD) 1, and PD-ligand 1, ICIs enhance T-cell activation, inadvertently promoting the recruitment of T-lymphocytes and macrophages to the myocardium, culminating in cardiomyocyte injury due to proposed molecular similarities between cardiomyocyte and tumor proteins, ultimately resulting in cell death and subsequent release of cardiac troponins. This specific inflammatory pathway drives the pathogenesis of ICI-related myocarditis and underpins the elevation of circulating troponin levels observed in clinical settings [12,49].

Serum troponin measurement plays a pivotal role in the diagnosis of ICI-associated myocarditis [8]. However, interpretation requires caution because ICI-related myocarditis frequently coexists with myositis. Previous studies report myositis in approximately 20–25% of patients with ICI-associated myocarditis [21], while myocarditis is present in up to 16.1% of patients with ICI-associated myositis [24]. Patients with skeletal muscle disease may exhibit elevated circulating cTnT concentrations even in the absence of myocardial involvement. Proposed mechanisms include cross-reactivity between skeletal muscle troponin T and cardiac troponin T assays, as well as aberrant re-expression of cTnT in diseased skeletal muscle. Although cTnT expression is normally suppressed after fetal development, it may be reactivated in skeletal myopathies, potentially contributing to falsely elevated circulating cTnT levels [15,22]. Consistent with these mechanisms, case reports have documented isolated cTnT elevation in patients with ICI-induced myositis without evidence of cardiac involvement. In contrast, cTnI does not appear to be similarly affected, suggesting that it may be a more specific biomarker for myocardial injury in this clinical setting [23,24,25]. A recent meta-analysis including 1,051 patients from four diagnostic studies demonstrated that troponin positivity, assessed using either cTnT or cTnI, was strongly associated with confirmed ICI-associated myocarditis; however, it did not compare the diagnostic performance of the two biomarkers [89]. Similarly, the 2022 ESC Guidelines on Cardio-Oncology acknowledge the clinical observation that cTnT may be falsely elevated in patients with myositis in the absence of myocarditis, but nevertheless recommend that either cardiac troponin assay may be used for the diagnosis of ICI-associated myocarditis [8].

Although ICI-associated myocarditis is an uncommon but potentially fulminant complication with reported mortality rates approaching 50% [12], cardiovascular toxicity extends well beyond myocarditis alone. Mann et al. demonstrated that atherosclerotic cardiovascular disease and heart failure account for most cardiovascular-related hospitalizations in patients receiving ICIs, highlighting their increased overall cardiovascular risk. Furthermore, patients with baseline hs-cTnT concentrations exceeding 14 ng/L experienced significantly higher rates of acute cardiovascular hospitalization and mortality. The combination of hs-cTnT and N-terminal pro B-type natriuretic peptide further improved risk stratification and identification of high-risk patients [88].

### 4.5. Endocrine Therapy

*Androgen deprivation therapy* (ADT), using gonadotropin-releasing hormone agonists or antagonists, is a key treatment modality for metastatic and locally advanced prostate cancer. However, cardiovascular toxicity is an important clinical concern, with some reports indicating an incidence of 20–30% among treated patients. In long-term prostate cancer survivors, cardiovascular disease has been proposed as the leading cause of mortality approximately 10 years after diagnosis [90,91,92]. Both experimental and clinical studies have reported elevations in cardiac troponins in association with ADT, although correlations with echocardiographic parameters or cardiovascular events have been inconsistent across studies [90,93,94,95]. Notably, Margel et al. reported an association between elevated baseline troponin levels and a higher risk of cardiovascular adverse events [95].

The molecular mechanisms underlying cardiac troponin release during ADT remain unknown. Testosterone deficiency has been associated with increased circulating cTnI levels and higher cardiovascular risk [95,96]. From a physiological perspective, testosterone is thought to exert cardioprotective effects by inhibiting cardiomyocyte apoptosis, stimulating protein synthesis, and improving left ventricular systolic and diastolic function [93]. In addition, androgens may reduce atherogenesis by limiting foam cell formation, attenuating endothelial injury, and suppressing inflammatory pathways [96]. Collectively, these mechanisms provide a plausible biological framework linking ADT-induced hypogonadism to subclinical myocardial damage and troponin release. It is possible that clinically relevant myocardial dysfunction develops only after prolonged ADT exposure, whereas cardiac troponins may reflect earlier subclinical injury and thus serve as early predictors of cardiotoxicity, analogous to their established role in anthracycline-associated cardiotoxicity, although further studies are required to clarify this relationship [90].

*Antiestrogen therapy.* Tamoxifen is the most widely used selective estrogen receptor modulator in the treatment of estrogen receptor-positive breast cancer. It functions as an estrogen receptor antagonist in breast tissue while exerting agonist activity in other tissues [97,98]. Although estrogen receptors in the cardiovascular system are generally associated with anti-inflammatory, anti-apoptotic, anti-atherogenic, vasodilatory, and angiogenic effects, clinical studies have demonstrated an association between tamoxifen therapy and adverse cardiovascular events. Accordingly, Meng et al. proposed that tamoxifen may exert antagonistic effects on estrogen receptor signaling in the heart. In a murine model, tamoxifen administration was associated with impaired left ventricular ejection fraction, increased serum cTnI levels, and cardiomyocyte apoptosis. Furthermore, based on experiments on cell cultures, Meng et al. suggested that myocyte injury was mediated through the IL-6/phosphorylated STAT3/peroxisome proliferator-activated receptor γ coactivator 1α/ROS signaling axis. This pathway was subsequently confirmed in mice [99] and may explain the observed elevation in cardiac troponin levels. However, clinical evidence supporting tamoxifen-associated troponin elevation remains limited. For instance, Silva et al. reported no increase in circulating cardiac troponin levels in patients treated with tamoxifen monotherapy [100].

### 4.6. Radiotherapy

Radiotherapy remains a fundamental component of cancer treatment; however, thoracic irradiation for breast, esophageal, lung cancers, and lymphomas carries a significant risk of cardiac injury. Although contemporary techniques have substantially reduced cardiac radiation exposure, radiation-induced heart disease remains an important long-term complication and may ultimately progress to heart failure [14,101,102,103]. hs-cTnT is a sensitive marker of subclinical myocardial injury during thoracic radiotherapy. Approximately 20–30% of patients develop hs-cTnT elevations compared with baseline, and these increases are dose-dependent, correlating with mean heart dose [101,102,103,104,105] and, according to one study, with left ventricular radiation dose [104]. Furthermore, Skytta et al. demonstrated that hs-cTnT elevation was associated with subtle echocardiographic alterations in left ventricular geometry and function. Although post-radiotherapy hs-cTnT concentrations generally remain below diagnostic thresholds for acute myocardial infarction, even small increases above 4 ng/L have been associated with adverse long-term cardiovascular outcomes in broader clinical populations [104].

Multiple mechanisms drive radiation-induced cardiomyocyte injury and subsequent troponin release, primarily involving microvascular dysfunction, profound oxidative stress, and the post-translational modification of contractile proteins [14,101,104,106]. The acute phase of cardiotoxicity is predominantly mediated by microvascular injury and tissue hypoxia. Ionizing radiation induces microthrombus formation within the myocardial capillary bed, resulting in downstream hypoxia-induced cardiomyocyte injury and the subsequent leakage of troponin into the circulation [104]. Concurrently, ionizing radiation directly disrupts mitochondrial respiration, promoting excessive ROS generation. Oxidative stress is further amplified by nitric oxide signaling and a robust inflammatory response involving macrophages, lymphocytes, and mast cells, ultimately leading to cardiomyocyte dysfunction and apoptosis [101]. Beyond conventional cell death pathways, radiation may promote troponin release through post-translational modification of contractile proteins. Rosen et al. identified irreversible oxidative carbonylation of cTnT as a potential radiation-specific mechanism. Carbonylation destabilizes the structural integration of cTnT within the sarcomere, facilitating its dissociation from the contractile apparatus and targeting the oxidized troponin for proteasomal degradation. Under conditions of sustained stress, structurally compromised cTnT could leak across the cardiomyocyte membrane into the bloodstream over the course of days, resulting in detectable troponin serum concentrations. Moreover, this mechanism could also lead to impaired contractile function [106].

## 5. Malignant Tumors Affecting the Myocardium

Beyond the cardiotoxic effects of cancer therapies, the tumor itself can directly–as in the case of cardiac metastases–or indirectly damage the myocardium, eventually leading to troponin elevation.

### 5.1. Direct Effect of Tumors on the Myocardium—Cardiac Metastases

Cardiac metastases represent a serious complication of advanced malignancies, with an incidence ranging from 0.7% to 18.3%, and are associated with a poor prognosis. Cardiac metastatic tumors arise through several distinct pathways, including direct infiltration from adjacent thoracic structures, hematogenous dissemination to the myocardium, and lymphatic spread. Certain malignancies demonstrate a distinct cardiac predilection for metastasis; for example, lung cancer more commonly involves the pericardium, whereas hepatocellular carcinoma more frequently metastasizes to the myocardium. Crucially, intracardiac (myocardial) metastases are associated with significantly higher troponin and B-type natriuretic peptide levels and poorer overall survival compared to isolated pericardial involvement. Therefore, unexplained elevations in troponin levels in patients with active malignancies—especially advanced lung, breast, or hepatic cancers—should immediately prompt clinicians to consider the possibility of cardiac metastasis [107,108]. Notably, a recent case series demonstrated that patients with myocardial metastases consistently exhibited elevated serum cTnT levels (26–98 ng/L), with further marked elevations observed in a case of superimposed myocardial infarction, highlighting the significant biochemical overlap between malignant infiltration and ischemic cardiac injury [108].

Elevated serum troponin in the setting of cardiac metastases can reflect multifactorial mechanisms of myocardial injury. Direct tumor infiltration into the myocardium causes localized ischemia and inflammation, triggering troponin release [12] (Figure 2). Additionally, tumors can induce ischemic injury through the extrinsic compression of epicardial coronary arteries or tumor embolization into the microvasculature [109,110].

Cardiac metastases can lead to diagnostic challenges as they can resemble myocardial infarction both clinically and biochemically. In cases of metastatic involvement, troponin elevations often lack the classic dynamic rise-and-fall pattern characteristic of coronary artery plaque rupture. Nevertheless, clinicians must recognize that these entities are not mutually exclusive; oncology patients can develop concomitant acute coronary syndrome due to plaque rupture or tumor-induced coronary occlusion [109,111]. Troponin elevation in patients with cardiac metastases is proposed to have prognostic significance. Higher serum troponin levels have been associated with shortened survival. Nevertheless, the correct interpretation of elevated troponin levels could aid in the early diagnosis of cardiac metastases, thereby rendering targeted therapeutic interventions possible [107,109].

### 5.2. Indirect Effects of Tumors on the Myocardium

Elevated hs-cTnT levels have been reported in patients with cancer independent of anticancer therapy or cardiac metastases. However, definitive cardiac pathologies are not always present in these individuals, and the underlying mechanisms remain incompletely understood [112]. One proposed explanation is that tumor tissue may directly contribute to a systemic inflammatory state and oxidative stress through the release of ROS and inflammatory cytokines, thereby promoting myocardial injury and subsequent troponin release [12,113,114] (Figure 2). This hypothesis may explain the observed elevation of cardiac troponin levels in cardiovascular disease-free, treatment-naive cancer patients; however, definitive evidence establishing a mechanistic link is still lacking.

*Paraneoplastic mechanisms.* In rare cases, malignancies can induce myocardial injury through paraneoplastic pathways [115,116,117]. For instance, clear cell renal cell carcinoma has been documented to trigger paraneoplastic dermatomyositis sine dermatitis with cardiac involvement characterized by elevated cTnI; however, this remains an exceptionally rare clinical manifestation [118].

*Coronary microvascular dysfunction* (CMD) is marked by impaired myocardial microcirculatory perfusion, resulting in insufficient oxygen supply, cardiomyocyte injury, and potential subsequent cardiac troponin release. CMD may result from inflammatory cytokine signaling (e.g., IL-1 and TNF-α), impaired vasodilation, microvascular spasm, and reduced capillary density, all of which may be induced by either the malignancy itself or anticancer therapies [12,119,120,121].

## 6. Clinical Implications

The expanding evidence on the mechanisms and causes of troponin release in cancer has important implications for cardio-oncology practice. Cardiac troponins have emerged as valuable biomarkers for identifying patients at increased risk of cardiovascular complications; however, their interpretation requires consideration of the unique pathophysiological processes associated with cancer, anticancer therapy and comorbidities. According to the current 2022 ESC Guidelines on Cardio-oncology, cardiac troponins play an important role in the diagnosis of cardiotoxicity, assessment of its severity, and close monitoring of patients who develop cancer therapy-related cardiovascular toxicity. They also support clinical decision making regarding the initiation of heart failure therapy and the continuation, interruption, or discontinuation of anticancer treatment [8].

*Risk stratification and prediction of cardiotoxicity.* Elevated baseline and on-treatment cardiac troponin concentrations consistently identify cancer patients at increased risk of all-cause mortality and cardiovascular mortality [7,10,11,122]. However, it remains unclear whether troponin elevation is associated with additional mortality risk specifically attributable to cancer or its treatment, or simply reflects the prognostic significance observed in the general population [3,123,124]. Beyond its prognostic value, early increases in troponin, particularly during anthracycline, trastuzumab, and radiotherapy, have repeatedly been shown to precede clinically overt cardiac dysfunction, supporting the roles of troponins as sensitive markers of subclinical myocardial injury and early risk stratification [29,48,125]. Accordingly, the 2022 ESC Cardio-Oncology Guidelines recommend baseline and serial troponin measurements in patients receiving certain cardiotoxic therapies, including anthracyclines, HER2 inhibitors, and immune checkpoint inhibitors, while the value of troponin measurement for many other anticancer agents remains to be established [8].

*Troponin assays and cut-off values.* The interpretation of troponin concentrations is complicated by differences in the kinetics of cTnI and cTnT [19], as well as the lack of oncology-specific cut-off values. Assay selection and the use of sex-specific versus sex-neutral upper reference limits may substantially influence the reported incidence of cancer therapy-related cardiac dysfunction [126]. Recent consensus documents support sex-specific upper reference limits, whereas current ESC guidelines do not explicitly recommend either approach [8,127]. Some authors have also proposed separate low- and high-risk thresholds rather than a single cut-off value [21].

Furthermore, the current ESC guidelines support the use of either cardiac troponin assay in clinical practice [8], as there is currently no evidence demonstrating the superiority of cTnT or cTnI in the oncological setting. The Class I, Level C recommendation for baseline troponin measurement is based on studies evaluating either cTnT or cTnI, including a meta-analysis by Michel et al. (cTnI: 3514 patients; cTnT: 1096 patients; both biomarkers: 594 patients) [2], studies by Lipshultz et al. (2012, cTnT, 156 patients; 2004, cTnT, 158 patients) [128,129], Xue et al. (hs-cTnT, 324 patients) [130], Cardinale et al. (2004, cTnI, 703 patients; 2010, cTnI, 251 patients) [79,131], Putt et al. (hs-cTnI, 78 patients) [132], and Zardavas et al. (both cTnT and cTnI, 452 patients) [133]. As these studies evaluated either cTnT or cTnI rather than directly comparing the two biomarkers, they do not allow conclusions regarding the superiority of one assay over the other for baseline cardiotoxicity risk prediction. Similarly, more recent evidence demonstrates the predictive value of both biomarkers. A meta-analysis by Lv et al. evaluating hs-cTnT (1294 patients) [29], a meta-analysis by Liu et al. evaluating cTnI (2033 patients) [48], and a study by Ju et al. evaluating hs-cTnI (415 patients) all demonstrated the prognostic value of troponins for predicting cardiotoxicity [125]. However, no studies have directly compared the predictive performance of cTnT and cTnI in cancer patients.

Another important consideration when interpreting cardiac troponin concentrations is the potential influence of patient ethnicity. Although not all studies have reached the same conclusion, several have demonstrated significant differences in cTnT and cTnI concentrations, as well as in their 99th percentile upper reference limits, among healthy individuals or cardiovascular disease-free populations of different ethnicities [134,135,136,137]. Moreover, studies investigating cancer therapy-induced cardiotoxicity have also reported significant differences between ethnic groups [138,139]. These observations suggest that ethnicity may likewise influence troponin levels in cardio-oncology patients; however, to date, no studies have directly investigated this question.

*Differential diagnosis.* Troponin elevation in cancer patients is not synonymous with cancer therapy-related cardiotoxicity. Besides treatment-induced myocardial injury, elevated troponin may result from acute myocardial infarction, chronic coronary artery disease, arrhythmias, anemia, systemic inflammation, renal dysfunction, or physiological stress [127,140]. Although high-sensitivity troponin assays remain highly sensitive for diagnosing acute myocardial infarction, the diagnostic accuracy and efficacy of conventional diagnostic algorithms may be reduced in patients with cancer, possibly owing to the frequent presence of elevated baseline troponin concentrations [141]. Additional causes, including skeletal muscle disease, presence of cardiac metastases, analytical interference (e.g., macrotroponin), and ectopic troponin expression by malignant tissue, should also be considered. At present, no widely applicable, dedicated diagnostic algorithm exists for interpreting troponin elevation in oncology patients, highlighting an important unmet clinical need [127].

## 7. Gaps in Evidence and Future Directions

Despite considerable progress, several important scientific gaps remain. Notably, the molecular mechanisms underlying troponin release in different oncological settings remain incompletely understood. Although several mechanisms have been identified experimentally, their relative contribution in humans remains uncertain. Future translational studies should identify the dominant mechanisms underlying troponin release across individual anticancer therapies and in cancer-associated cardiac injury. Moreover, as the mechanisms responsible for cancer-associated cardiac injury and subsequent troponin release remain uncertain, the clinical and biochemical characteristics that may help clinicians differentiate between cancer therapy-related and malignancy-associated troponin elevation have yet to be established. Similarly, current evidence is insufficient to comprehensively compare anticancer treatment modalities regarding the timing and magnitude of troponin elevation, its association with left ventricular dysfunction, reversibility, prognostic significance, and optimal monitoring strategies, highlighting an important area for future research. In addition, although numerous studies have identified cardiac troponin as an independent predictor of mortality in oncology populations, it remains unclear whether troponin confers additional prognostic information beyond that observed in the general population. Furthermore, no oncology-specific cut-off values currently exist for either cTnT or cTnI, as thresholds have been derived from general cardiology populations. Also, the relative clinical utility of cTnT and cTnI remains unsettled, particularly considering their differing release kinetics, sensitivity to comorbidities, and susceptibility to interference from skeletal muscle disease and macrotroponin. Further studies are needed to determine whether, in specific clinical scenarios, including suspected ICI-myocarditis with concomitant myositis, cTnT or cTnI may provide superior diagnostic value, and whether simultaneous measurement of both biomarkers could offer additional diagnostic information in selected settings. Another unresolved question is whether racial or ethnic differences in troponin levels exist among cardio-oncology patients. Finally, with the exception of a few cardiotoxic agents, prospective studies evaluating troponin-guided surveillance, cardioprotective therapy, treatment modification, and individualized monitoring strategies remain scarce.

Future investigations should therefore address these gaps in evidence. As multiple assays and different cut-off values exist for both cTnT and cTnI, standardization is required not only in clinical practice, but also in research to ensure comparability across future studies. Finally, although the role of troponin is relatively well established in anthracycline-, anti-HER2 therapy-, and immune checkpoint inhibitor-associated cardiotoxicity, its clinical utility in other anticancer treatment settings remains poorly explored. Therefore, future research should focus on developing treatment-monitoring protocols as well as a comprehensive, validated algorithm for the differential diagnosis of troponin elevation in broader cohorts of cancer patients.

*Potential ectopic expression of cardiac troponins.* Recent studies have challenged the paradigm that cardiac troponin expression is restricted exclusively to cardiomyocytes by demonstrating the presence of cardiac troponin isoforms in malignant tissues. Ectopic cTnI and cTnT expression has been reported in lung and colorectal cancer tissues [113,142,143,144,145]. In lung cancer, cardiac troponin expression was associated with advanced-stage, metastatic, or recurrent disease in a study by Tsuruda et al. [113], while experimental models suggested that cTnI or cTnT overexpression may promote tumor cell proliferation, migration, and colony formation through activation of pathways including Kras, Notch, ErbB, and epidermal growth factor receptor-related signaling [143,144,145]. Similarly, in colorectal cancer, increased TNNT2 expression was associated with advanced disease stage, poorer prognosis, and enhanced tumor cell aggressiveness [144]. The potential clinical relevance of ectopic tumor-derived troponin expression was highlighted by Tsuruda et al., who described a patient with metastatic ethmoid sinus carcinoma and markedly elevated cTnT levels following nivolumab therapy despite the absence of significant cardiac abnormalities. Immunohistochemical analysis demonstrated cTnT expression in the cytoplasm and nucleus of tumor cells, suggesting that circulating troponin may have originated from tumor cell injury or turnover rather than myocardial damage [146]. These findings indicate that cardiac troponins may have previously unrecognized roles beyond cardiomyocytes and may contribute to tumor biology. However, whether ectopic tumor-derived troponin expression contributes to elevated circulating troponin levels remains uncertain, with only a single case report directly linking serum troponin elevation to tumor expression. Future studies should investigate the clinical relevance of this phenomenon and determine whether ectopic cardiac troponin expression should be considered when interpreting elevated troponin levels in selected oncological scenarios.

## 8. Conclusions

Cardiac troponin has become a key biomarker in cardio-oncology, extending far beyond its traditional role in the diagnosis of acute myocardial infarction. As summarized in this review, elevated circulating troponin concentrations in patients with cancer arise through diverse mechanisms, including classical necrosis, as well as regulated cell death pathways such as apoptosis, necroptosis, pyroptosis, and ferroptosis, each contributing to varying degrees across specific anticancer therapies, including anthracyclines, anti-HER2 agents, tyrosine kinase inhibitors, immune checkpoint inhibitors, and radiotherapy [12,38,49,106]. Beyond therapy-induced injury, malignancies themselves may increase circulating troponin concentrations through cardiac metastases or indirect systemic effects [107,112,118].

Importantly, the significance of cardiac troponin in oncology extends beyond its role as a biomarker of myocardial injury. Recently it has been demonstrated that certain malignancies, including lung and colorectal cancer, can aberrantly express cardiac troponin isoforms, with experimental studies suggesting that this ectopic expression actively promotes tumor proliferation and invasiveness [144,146]. Furthermore, some studies propose that certain anticancer therapies impair myocardial contractility through direct effects on sarcomeric troponin [86,106], although the translational implications of these mechanistic advances remain to be explored.

Across multiple cancer populations, elevated baseline or treatment-related troponin concentrations consistently identify patients at increased risk of cancer therapy-related cardiac dysfunction, major adverse cardiovascular events, and mortality, supporting the incorporation of troponin measurements into contemporary cardio-oncology surveillance strategies [8,48,88,125]. However, interpretation of troponin elevation in oncology remains challenging because circulating concentrations may reflect multiple concurrent processes, including treatment-related myocardial injury, systemic inflammation, ischemic heart disease, renal dysfunction, skeletal muscle disease, or tumor-associated mechanisms [112,127,140]. Collectively, these findings highlight that troponin in cardio-oncology is a multifaceted biomarker whose interpretation depends on treatment modality, tumor biology, and patient-specific comorbidities.

## Data Availability

No new data were created or analyzed in this study. Data sharing is not applicable to this article.

## Abbreviations

The following abbreviations are used in this manuscript:

ADT	Androgen deprivation therapy
cTnC	Cardiac troponin C
cTnI	Cardiac troponin I
cTnT	Cardiac troponin T
eNOS	Endothelial nitric oxide synthase
ESC	European Society of Cardiology
HER	Human epidermal growth factor receptor
hs-	High sensitivity-
ICI	Immune checkpoint inhibitor
PI3K	Phosphatidylinositol 3-kinase
ROS	Reactive oxygen species
STAT	Signal transducer and activator of transcription
TKI	Tyrosine kinase inhibitor
TNF	Tumor necrosis factor
VEGF-A	Vascular endothelial growth factor A
